# A Case Report of Mixed Osteomalacia and Low Bone Density from Vitamin D Deficiency as a Cause of Bilateral Tibial Stress Fractures in a Young Male Military Recruit from Singapore

**DOI:** 10.1155/2020/9519621

**Published:** 2020-01-24

**Authors:** Wann Jia Loh, Louise Hughes, David Thai Chong Chua, Linsey Gani

**Affiliations:** ^1^Department of Endocrinology, Changi General Hospital, 2 Simei Street, Singapore 529889; ^2^Department of Anatomical Pathology, Concord Hospital, Hospital Road, Concord West, New South Wales 2139, Australia; ^3^Department of Orthopaedics, Changi General Hospital, 2 Simei Street, Singapore 529889

## Abstract

Despite being a tropical country, vitamin D deficiency is common in Singapore. All young Singaporean males between the age of 18 and 21 years have to undergo mandatory military service. Stress fractures occur in military recruits, and risk factors include a sudden increase in physical activity and vitamin D deficiency. We report the bone histomorphometry findings from a case of bilateral tibial stress fractures in an 18-year-old military recruit who had vitamin D deficiency. The histomorphometry showed a mixed osteomalacia and osteoporosis pattern. This case is unique as it shows that stress fractures from a marching exercise can occur in apparently healthy well young man with vitamin D deficiency despite living in a tropical country.

## 1. Introduction

Stress fractures in military recruits are not uncommon with rapid increase in physical activity and low vitamin D level being major risk factors [1–3]. Despite a tropical country, vitamin D deficiency is common in Singapore [4, 5]. All young Singaporean males between the age of 18–21 years have to undergo 2 years of mandatory military service. We describe the bone histomorphometry finding of an 18-year--old military recruit who sustained bilateral tibial fractures in his initial basic military training course and findings of a mixed osteomalacia and low bone density with improvement in his bone density after 2 years of adequate vitamin D replacement.

## 2. Case Presentation

Our patient is an 18-year-old man of Chinese ethnicity who presented with bilateral leg pain after a short march of 200 meters during his first marching session. He has no other significant past medical history, in particular he had completed his puberty normally, and there was no previous history of GI malabsorption. There was no history of any long-term medication administration. There was no family history of osteoporosis or fractures. He did not smoke or drink alcohol. His body mass index (BMI) was 24.6 kg/m^2^ (weight 73.5 kg, height 1.73 m). Further history revealed that the acute severe bilateral leg pain started after taking part in a marching session of 200 meters during his 1^st^ week of basic military training. Initially, he had pain in his left shin after the march, and this was followed by pain in his right shin a day later. Because of the pain, he was unable to walk. The march was not a particularly stressful exercise, and he was not carrying any weights. However, this was his first marching session, and he has always been very physically inactive throughout his childhood and does not exercise regularly.

**Figure 1:**
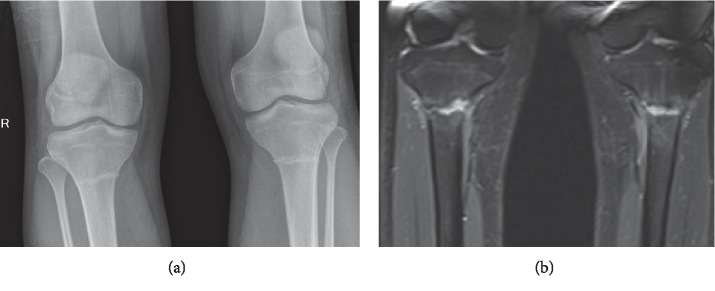
X-ray and MRI. (a) X-ray of the patient's legs showed cortical irregularity and lucency with sclerotic margins in the proximal shaft of the tibia, confirming stress fractures. (b) MRI of the calves of the patient showed increased signal at linear T1RM sequence which suggests fluid clefs at both fracture sites.

On examination, there was tenderness on palpation of the both proximal tibias. There were no blue sclerae or hypermobile joints. He was at Tanner stage 5 for puberty. An X- ray of his legs showed cortical irregularity and lucency with sclerotic margins in the proximal shaft of the tibia, confirming stress fractures (Figure 1(a)). The radiograph did not show features of bowing. Magnetic resonance imaging (MRI) of both calves confirmed transverse fractures of the bilateral proximal tibial metaphysis associated with bone marrow oedema and periosteal reaction (Figure 1(b)). His bone mineral density (BMD) using dual energy X-ray absorptiometry (DEXA) showed that he had lower bone mass and density expected for his age; his BMD of the lumbar spine measured 0.751 g/cm^2^, with a corresponding *Z* score of −2.4. The BMD of the left hip measured 0.847 g/cm^2^, corresponding to a *Z* score of −1.8; whereas, the BMD of the left femoral neck was 0.789 g/cm^2^, corresponding to a *Z* score of −1.5. Secondary causes of osteoporosis were investigated for and were negative apart from a low vitamin D level of 10.7 *μ*g/L (Table 1). Although coeliac screening is part of young osteoporosis workup, coeliac screening was not performed because he did not have predilection for autoimmune disease and the rarity of this disease in a person of Chinese ethnicity. The chest radiograph was normal. The X-ray of the pelvis did not show any features of hip deformity seen in osteomalacia such as coxa profunda or coxa vara.

**Figure 2:**
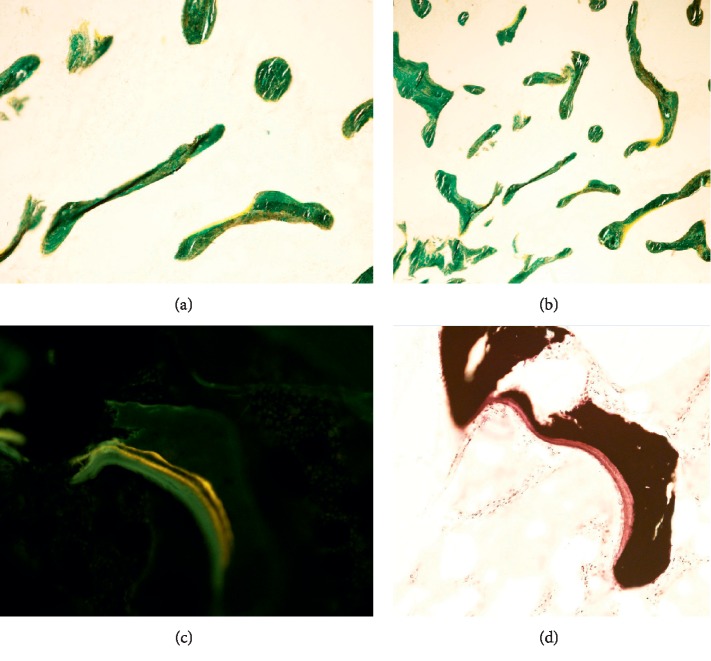
Bone biopsy. (a) High resolution (alkaline phosphatase staining). (b) Low resolution (alkaline phosphatase staining). Both low and high power showed irregularity, variability, and loss of connectivity of bone trabeculae, with some thinning and scattered bone nubbins. (c) Unstained bone biopsy. (d) The Von Kossa (calcium) stain showed the thickened osteoid seam (red) with the calcified bone (black).

Double tetracycline labelled qualitative bone histomorphometry was performed from the iliac crest with administration of tetracyline at day 1, 2, 15, and 16 followed by a bone biopsy at day 22. The results showed delay in mineralization, with increase in osteoid and blurred tetracycline labelling, as seen in osteomalacia (Figure 2). However, unlike osteomalacia, there was not a significant increase in the number of osteoblasts. There was also some degree of variability, thinning, and loss of connectivity of trabeculae (Figure 2), which are seen in osteoporosis. This was significant for his age. Therefore, the bone biopsy suggested a mixed picture of osteomalacia and osteoporosis. He was treated conservatively, given vitamin D supplementation, and advised adequate calcium intake.

### 2.1. Outcome and Follow-Up

With vitamin D replacement, the vitamin D level of the patient improved from 10.7 to 41.4 *μ*g/L after 3 months. He was continued on maintenance vitamin D3 cholecalciferol 1000 international units per day to maintain a vitamin D level of >30 *μ*g/L. Two years later, a repeat BMD performed using the DEXA scan showed significant improvement of BMD; 5.4% increase in the lumbar spine which represents a significant change (least significant change (LSC) = 0.22 g/cm^2^) and 3.2% increase in BMD of the hip, which does not represent a significant change (LSC = 0.034 g/cm^2^). He has not sustained any further fractures for the last 3 years since then.

## 3. Discussion

Despite being a tropical country (1°18′N 103°51É with mean sunshine 2022.4 h/y), vitamin D deficiency is prevalent. A previous study found 42.1% of young healthy adults in Singapore were found to have vitamin D levels of less than 20 ng/ml, although its prevalence in females was higher [1]. This in combination with increasing lack of physical activity in the young may herald an increase in the prevalence of poor bone health in the population [2]. Our case report demonstrates histology-proven osteomalacia due to vitamin D deficiency in an otherwise healthy young man. Of note, he has had no previous fractures, and his investigations did not reveal typical radiological abnormalities of osteomalacia. This emphasizes the importance of vitamin D in the maintenance of bone health especially in the young.

Osteomalacia is a disorder of bone characterized by delayed mineralization of newly formed osteoid at sites of bone turnover. Several different disorders may cause vitamin D deficiency leading to osteomalacia such as gastrointestinal malabsorption, liver disease, and renal disease [3]. Osteomalacia may be asymptomatic and present radiologically as osteopenia and Looser zones. Symptoms include bone pain, muscle weakness, fracture, and muscle cramps [3]. Osteomalacia is believed to be rare in developed nations due to the adequate nutrition, and previous cases of osteomalacia reported in the literature are mostly in the elderly or those with gastrointestinal malabsorption [3]. In this case, the bone histomorphometry showed that although there was delay in mineralization, unlike in osteomalacia, there was not a significant increase in the number of osteoblasts. We hypothesized that this may be due to the delay in bone biopsy which was performed about 6 weeks after the fracture, and the patient was already commenced on vitamin D replacement. Hence, the appearance of osteomalacia on bone histomorphometry may be attenuated.

The incidence of stress fractures has been reported to be as high as 20% in runners and accounts for 10–20% of consultations in a sports medicine clinic [4]. While the true incidence of stress fractures of the military recruits in our country is unknown, it is worthwhile to note that the incidence of stress fractures in U.S. military recruits was estimated to be 38 to 77 per 1000 person-years in 2009 [4] and reported to occur in 7.2% of UK Royal Marine military recruits [5]. The most common site of stress fractures is the tibia, followed by tarsals and metatarsals [6]. Bilateral femoral fractures have also been reported in young military recruits [4, 7]. While most stress fractures are uncomplicated and can be managed conservatively, there are some with high-risk stress fractures that have delayed recovery, chronic pain, and increased risk of progression to complete fracture. This usually affects specific sites which have a maximal tensile load in regions of hypovascularity and hence potentially reduces healing such as femoral neck, patella, anterior tibia, and medial malleolus [6]. Stress fractures occur more commonly at cortical bone instead of cancellous bone likely because of the slower remodeling rate of cortical bone [6].

The pathogenesis of stress fractures is suspected to be due to the imbalance between bone resorption and bone formation, in the setting of repeated mechanical loading without adequate rest. This leads to microfractures, bone oedema, and subsequently stress fractures [6].

The known risk factors predisposing to stress fractures include female gender, vitamin D deficiency, secondary causes of osteoporosis (e.g., malabsorption and hyperthyroidism), previous stress fracture, and excessive training intensity, frequency, or duration [5, 6]. Females are at higher risk than men to suffer from stress fractures among both runners (9.2% in females and 3% in males) and military recruits (9.7% vs 6.5%, respectively) [8]. This may be explained by their smaller muscle size, dietary difference, and bone anatomy [8]. Vitamin D deficiency also increases the risk of stress fractures [9]. In a study of 1082 Royal Marine military recruits, vitamin D deficiency below 20 *μ*g/L has been associated with increased risk of stress fractures in military recruits [5]. It is often misconceived that a tropical country has low rates of vitamin D deficiency. Recent studies reported that vitamin D deficiency (<20 *μ*g/dL) was present in 13.4% of pregnant women [9] and 42% of healthy individuals [1]. In Singapore, the enrollment for compulsory national service is 18 years old which is a critical time of accruing bone mass, as peak bone mass range from early to late 20s [10].

Interventions to identify high-risk individuals and prevent fractures can be successful. For example, the U.S. Armed Forces successfully reduced the stress fracture rate by almost 60% by identifying individuals with slower run times, reducing frequency of running in less-fit individuals, and ensuring that every trainee receives adequate nutrition including vitamin D and calcium [4]. Individuals with vitamin D deficiency should be replaced with adequate vitamin D to ensure peak bone mass and may benefit from higher vitamin D load (e.g., 2000IU) [11] although clinical evidence for this is lacking. Our patient showed improvement of his bone mineral density with vitamin D repletion. Further studies are required to show if vitamin D supplementation at a younger age may help to ensure adequate peak bone mass, prior to the enlistment for national service.

## 4. Conclusion

This case demonstrates the importance of vitamin D repletion and possibility of osteomalacia presenting in an otherwise healthy young patient without typical symptoms, biochemical, and radiology findings due to inadequate vitamin D. Further studies should look into regular vitamin D supplementation especially in young patients with previous minimal sunlight exposure and regular physical activity who are entering into military service.

## Figures and Tables

**Table 1:** The summary of investigations performed to evaluate secondary causes of osteoporosis.

Blood tests	Patient	Reference range
Free thyroxine (pmol/L)	12.16	10–20
Thyroid stimulating hormone (mIU/L)	0.733	0.4–4
Albumin (g/L)	39	37–51
Haemoglobin (g/dL)	12.8	13–17
Creatinine (*μ*mol/L)	59	65–125
25-Hydroxy vitamin D (*μ*g/L)	10.7	
Calcium (mmol/L)	2.23	2.1–2.6
Phosphate (mmol/L)	1.3	0.65–1.65
Parathyroid hormone (pmol/L)	6.55	1.3–7.6
Alkaline phosphatase (U/L)	94	32–103
Total testosterone (nmol/L)	11.65	9.9–27.8
24-Hour urine free cortisol (nmol/day)	245	59–413
Urine calcium (mmol/L)	0.3	<0.6
